# The Associations of Dietary Iron, Zinc and Magnesium with Metabolic Syndrome in China’s Mega Cities

**DOI:** 10.3390/nu12030659

**Published:** 2020-02-28

**Authors:** Zhenni Zhu, Yuna He, Fan Wu, Liyun Zhao, Chunfeng Wu, Ye Lu, Jiajie Zang, Zhengyuan Wang, Jing Sun, Jian Huang, Changyi Guo, Gangqiang Ding

**Affiliations:** 1 Division of Health Risk Factors Monitoring and Control, Shanghai Municipal Center for Disease Control and Prevention, 1380 West Zhongshan Road, Shanghai 20036, China; zhuzhenni@scdc.sh.cn (Z.Z.); wufan@scdc.sh.cn (F.W.); wuchunfeng@scdc.sh.cn (C.W.); zangjiajie@scdc.sh.cn (J.Z.); wangzhengyuan@scdc.sh.cn (Z.W.); guochangyi@scdc.sh.cn (C.G.); 2 National Institute for Nutrition and Health, Chinese Center for Disease Control and Prevention, 27 Nanwei Road, Xicheng District, Beijing 100050, China; heyn@ninh.chinacdc.cn (Y.H.); zhaoly@ninh.chinacdc.cn (L.Z.); sunjing@ninh.chinacdc.cn (J.S.); huangjian@ninh.chinacdc.cn (J.H.); 3 Division of Non-Communicable Diseases Prevention and Control, Shanghai Municipal Center for Disease Control and Prevention, 1380 West Zhongshan Road, Shanghai 20036, China; luye@scdc.sh.cn

**Keywords:** dietary, iron, zinc, magnesium, metabolic syndrome, food sources

## Abstract

Background: Iron, zinc and magnesium perform differently in body metabolism but exist in similar food. This study was to evaluate the associations of dietary iron, zinc and magnesium with metabolic syndrome (MetS). Methods: A sample of a total of 5323 participants from four of China’s mega cities was included in the current study. Both a 3-day 24-h dietary recall and household condiment weighing were applied to assess dietary intake, respectively. Hierarchical logistic regression models were used to evaluate the associations of dietary iron, zinc and magnesium with MetS. Results: After adjusting for age, sex, region, years of education, physical activity level, intended physical exercises, smoking status, alcohol use, daily energy intake and mutual adjustment for dietary iron, zinc and magnesium, significant positive trends were found across quartiles of total dietary iron and the risk of MetS, as well as for magnesium and MetS (*p* value for trends = 0.01 and 0.02, respectively); dietary zinc was inversely associated with MetS risk (*p* value for trend < 0.01). Magnesium from grains and potato was positively associated with MetS (*p* value for trend < 0.01). Conclusions: Dietary iron and magnesium were positively associated with the risk of MetS, while zinc was inversely associated with the risk of MetS, in China’s mega cities. The positive association of magnesium with MetS could be a result confounding by other factors correlated with magnesium in grains and potato, which warrants further study.

## 1. Introduction

Metabolic syndrome (MetS) refers to a constellation of interrelated risk factors that increase the development of both cardiovascular disease and type 2 diabetes mellitus [1]. The nationwide prevalence of MetS among Chinese adults has remarkably increased from 9.8% to 24.2% within one decade [2,3], which poses a large health threat to the Chinese population.

Iron is an essential element for cell growth, erythropoietic function, oxidative metabolism and cellular immune responses [4]. However, excess iron can cause tissue damage or subclinical inflammation. Free iron has strong pro-oxidant properties and generates reactive oxygen species by participating in Fenton chemistry, which results in the induction of oxidative damage and apoptosis. Some studies have suggested that a greater dietary iron intake is associated with the development of MetS [5,6,7]. Zinc is essential to many metabolic processes [8]. It acts as an antioxidant, has membrane-stabilizing properties, blocks apoptotic cell death and is essential for endothelial integrity [9,10]. Therefore, zinc probably plays a favorable role in the prevention of metabolic syndrome [11]. Magnesium serves as a cofactor or activator in more than 600 enzymes and influences extracellular calcium levels. Its deficiency might play a central role in the development of MetS [12].

Though iron, zinc and magnesium perform differently in body metabolism, they are found in similar food sources and are positively associated with each other [13,14,15]. In addition, some studies show iron and zinc from different food sources do not consistently play the same role in the association with MetS [6,16]. Iron deficiency has been long kept in focus in China due to the poor iron bioavailability of well-known Chinese plant-based diets. However, dietary patterns have transited into “rich” patterns in Chinese mega cities which feature far less plant food and excessive animal food. The primary objective of this study was to evaluate the associations of dietary iron, zinc and magnesium with MetS. Furthermore, this study also aimed to identify the correlations between dietary sources of the minerals mentioned above and MetS.

## 2. Materials and Methods

### 2.1. Study Population

The data used in this study were obtained from two independent cross-sectional investigations with high homogeneity. One was China Nutrition and Health Survey (CNHS) funded by national finance. The other was the Shanghai Diet and Health Survey (SDHS) funded by provincial finance. The data of four mega cities in China (the exclusive four municipalities directly under the Central Government of China, which are Beijing, Shanghai, Tianjin and Chongqing.) were included in the current analysis. The data of Beijing, Tianjin and Chongqing were collected during 2010–2012 by CNHS, and the data of Shanghai were obtained in 2012 by SDHS. The two surveys were designed to identify the dietary intake and nutritional status of the population. Community-dwelling residents and their family members, aged at least 18 years, were selected though a random multi-stage stratified sampling.

**Figure 1:**
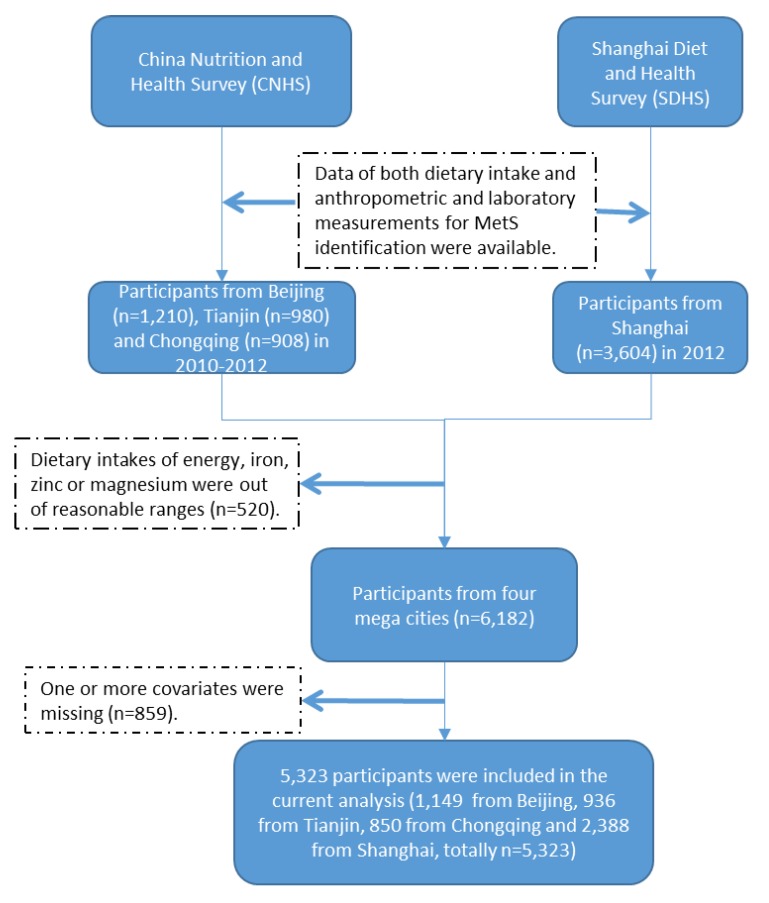
Participants flow chart.

The initial eligible data of both dietary intake and anthropometric and laboratory measurements for MetS identification were 3098 records (1210 from Beijing, 980 from Tianjin, and 908 from Chongqing) from CNHS and 3604 records from SDHS. Mean ± 2SD was applied to setting reasonable ranges of energy, iron, zinc and magnesium intake in the study population. A total of 520 participants were excluded for dietary intakes of energy, iron, zinc or magnesium without the reasonable ranges. The reasonable ranges were set as daily energy intake from 800kcal to 4500kcal, iron less than 40mg, zinc less than 20mg, and magnesium less than 450mg, respectively. Another 859 participants were excluded due to having one or more covariates missing. A final sample of total 5323 participants were determined in the current analysis; 1149 from Beijing, 936 from Tianjin, 850 from Chongqing and 2388 from Shanghai. (Figure 1)

The CNHS was approved by the ethics committee of the National Institute for Nutrition and Health at the Chinese Center for Disease Control and Prevention. The SDHS was approved by the Shanghai Municipal Center for Disease Control and Prevention’s Institutional Review Board. Informed consent was obtained from each participant before the survey. The study complied with the code of ethics of the World Medical Association (Declaration of Helsinki).

### 2.2. Dietary Assessment

The same dietary survey methods were applied in CNHS and SDHS including a 3-day 24-h dietary recall for 3 consecutive days (including 2 weekdays and 1 weekend day) and 3-day household condiments weighing. The interviewers were public health doctors from local centers of disease control and prevention and community health centers. They received a standard training course on the recording of dietary information. Each participant was orally instructed to record his/her daily food intake both at home and out of home on draft paper at the beginning and interviewed face-to-face by interviewers in the consecutive survey days at home. At each survey day, the interviewers collected and checked though the draft paper, and afterward, revised the food weight and transcribed the draft dietary information into a structured form. Household condiments mainly containing fat or sodium, including cooking oil, salt, soy sauce and chili sauce, were weighed before and after the 3 survey days. Furthermore, the participants were instructed not to change their typical diet or life style during the survey period. No disastrous events, such as rain or snow disasters, that would have affected the normal food supply occurred during the survey period.

Daily food consumption was calculated from the 3-day 24-h dietary recall. The 3-day consumption of condiments based on the calculated weight difference was divided into individual intake according to the eating times in the home and the individuals’ energy intake (only from food) proportion among family members. The consumption of condiments from meals out of home was simulated according to the previously calculated condiments’ densities in food consumed at home. The intakes of dietary energy, iron, zinc and magnesium were estimated according to daily food and condiment consumption using the Chinese food composition database [17,18]. There is no compulsory fortification of food in China, except iodized salt. Since only 0.7% of Chinese were using nutrient supplements [19], dietary supplements and medications were excluded from nutrient intake. Heme iron was estimated as 40% of the total iron from animal foods, including red meat, poultry, fish, and animal organs, and non-haem iron was calculated as the remaining portion of the total iron from all foods [20,21].

### 2.3. Potential Confounders

The information other than dietary intake including each participant’s age, sex, region, years of education, physical activity level, intended physical exercises, smoking status and alcohol use was obtained in both CNHS and SDHS. The educational level of the participants was reported as years of education. The physical activity level was recorded as sedentary, moderate, or vigorous according to professional activities. Intentional physical exercise was defined as physical exercise performed for the purpose of health maintenance or fitness. Smoking status was categorized as never smoked, former smoker or current smoker. With respect to alcohol use, the respondents were classified as follows according to their alcohol consumption: Lifetime abstainers, the respondent did not consume an alcoholic beverage; nonheavy drinkers (social drinkers), the respondent consumed 50g alcohol for male and 40g for female once (1 day) in the 1-week period; infrequent heavy drinkers, the respondent consumed 50g alcohol for male and 40g for female on 2–3 days in the 1-week period; frequent heavy drinkers, the respondent consumed 50g alcohol for male and 40g for female on at least 4 days in the 1-week period.

### 2.4. Anthropometric and Laboratory Measurements

Anthropometric measurements in CNHS and SDHS were under the same standard criteria. These were conducted in the community health centers located in each participant’s community. Waist circumference was measured using a tape measure. Blood pressure was measured three times after a quiet rest for 5 min.

Each participant was asked to fast for more than 10 h, and their blood was then collected to measure the serum concentrations of glucose, triglycerides, high-density lipoprotein-cholesterol (HDL-C) and ferritin. The blood samples from Beijing, Tianjin and Chongqing were analyzed at the laboratory of the National Institute for Nutrition and Health, Chinese Center for Disease Control and Prevention and those from Shanghai were at the laboratory of the Shanghai Municipal Center for Disease Control and Prevention. Serum glucose, triglycerides and HDL-C were assayed on a HITACHI 7080 or 7600 Automatic Biochemical AnalyzerThe reagents were all from Wako Pure Chemical Industries, Ltd. (Tokyo, Japan). Serum ferritin was measured by BECKMANCOULTER ACCESS II Automatic Chemiluminescent Immunoassay Analyzer.2.5 Definition of Metabolic Syndrome

MetS was identified based on the criteria in the US National Cholesterol Education Program Adult Treatment Panel III (NCEP-ATP III) for Asian populations [1], which states that at least three of the following metabolic abnormalities should be present: (1) Elevated waist circumference (WC ≥ 90 cm for men and WC ≥ 80 cm for women); (2) elevated triglycerides (triglycerides ≥ 150 mg/dL) or on drug treatment for elevated triglycerides; (3) reduced HDL-C (HDL-C < 40 mg/dL for men and < 50 mg/dL for women) or on drug treatment for reduced HDL-C; (4) elevated blood pressure (systolic blood pressure ≥ 130 mmHg and/or diastolic blood pressure ≥ 85 mmHg) or on antihypertensive drug treatment with a history of hypertension; and (5) elevated fasting glucose (100 mg/dL) or on drug treatment for elevated glucose.

### 2.5. Statistical Analyses

Statistical analyses were conducted using SAS statistical software (v. 9.2; SAS Institute, Cary, NC, USA). Since the similar genetic background in families might lead to the aggregation of MetS occurrence, hierarchical logistic regression models were introduced in the analysis of determining the odds ratios (ORs) and 95% confidence intervals (CIs) of the occurrence of MetS based on four tertiles of iron, zinc and magnesium intake. The mineral intakes were used as the independent fixed-effect variables and the family aggregation was used as the random-effect variable. A two-sided *p* value < 0.05 was considered to indicate statistical significance.

## 3. Results

### 3.1. Characteristics of the Participants and Correlations of Dietary Iron, Zinc and Magnesium Intakes

The analysis sample included 5323 participants, consisting of 45% male adults and 55% female adults. Participants aged 18–44 years, 45–59 years and 60+ years were 26.22%, 38.56% and 35.22%, respectively. The average dietary total iron, zinc and magnesium intakes were 17.65 ± 6.18 mg/day, 9.17 ± 3.21 mg/day and 253.27 ± 98.74 mg/day, respectively. The prevalence of MetS among the study population was 34.49%. The detailed characteristics of the participants were shown in Table 1. Age- and sex-adjusted correlations between dietary iron, zinc and magnesium intakes ranged from 0.71 to 0.82 (Table 2). Dietary iron, zinc and magnesium intakes were highly correlated with each other.

### 3.2. The Associations of Dietary Iron, Zinc and Magnesium with MetS

After adjusting for age, sex, region, years of education, physical activity level, intended physical exercises, smoking status, alcohol use, daily energy intake and mutual adjustment for iron, zinc and magnesium, a significant positive trend was found across quartiles of dietary total iron and the risk of MetS (*p* value for the trend = 0.01). Compared with the lowest quartile of dietary total iron intake (< 13.14 mg/day), the highest quartile (≥ 21.41 mg/day) had an OR (95% CI) of 1.60 (1.21, 2.11). The trend between non-haem iron and MetS was similar with that between total iron and MetS, while the trend between haem iron and MetS was on the contrary. There was a negative trend between increasing quartiles of dietary zinc and MetS risk (*p* value for the trend < 0.01). The highest quartile of zinc intake (≥ 11.19 mg/day) had nearly a half lower risk of MetS than the lowest quartile (< 6.87 mg/day). A significant positive trend was observed between dietary magnesium and MetS risk (*p* value for the trend = 0.02). The highest quartile of magnesium intake (≥ 304.34 mg/day) had 1.32-folded risk of MetS than the lowest quartile (< 182.98 mg/day). (Table 3)

Dietary iron intake was correlated with serum ferritin, an indicator of body iron storage, independent of age and sex in the study population (Supplement Table S1). Sex differences were not observed in the associations of dietary iron, zinc and magnesium with MetS in the current participants (Supplement Table S2).

### 3.3. The Associations of Dietary Sources of Iron, Zinc and Magnesium with MetS

**Figure 2:**
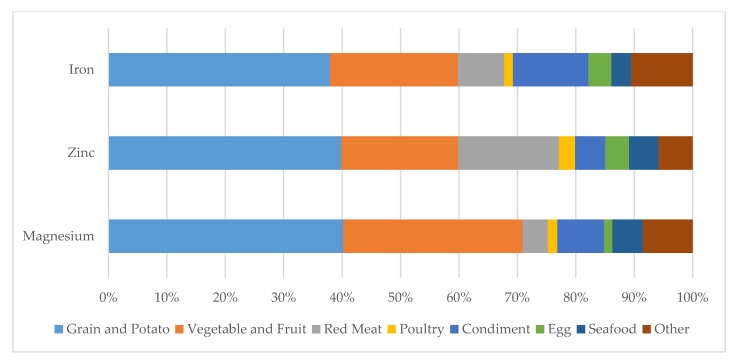
Dietary sources of iron, zinc and magnesium among 5323 participants from CNHS and SDHS.

The major dietary sources of iron, zinc and magnesium were grains and potato, vegetables and fruit, and red meat in the study population from China’s mega cities (Figure 2).

After adjusting for age, sex, region, years of education, physical activity level, intended physical exercises, smoking status, alcohol use and daily energy intake (Model 2), iron from red meat, as well as from vegetables and fruit, was negatively associated with MetS risk, while iron from grains and potato was positively associated (*p* value for the trends < 0.01, < 0.01 and = 0.01, respectively). After further mutually adjusting for iron, zinc and magnesium from the same food sources (Model 3), the trends were no more statistically significant, and the ORs across the increasing quartiles of iron from red meat and grains and potato turned closer to 1(Table 4).

After adjusting for age, sex, region, years of education, physical activity level, intended physical exercises, smoking status, alcohol use and daily energy intake (Model 2), zinc from red meat, as well as from vegetables and fruit, was negatively associated with MetS risk, while zinc from grains and potato was positively associated (all *p* values for the trends < 0.01). After further mutually adjusting for iron, zinc and magnesium from the same food sources (Model 3), the trends between zinc from red meat and MetS risk, as well as between zinc from vegetables and fruit and MetS risk, were no more statistically significant, while the trend between zinc from grains and potato and MetS risk was inverse. Zinc from grains and potato was negatively associated with MetS risk (*p* value for the trend = 0.01). The ORs for MetS risk across the increasing quartiles of zinc from vegetables and fruit turned closer to 1, but the ORs across the quartiles of zinc from red meat remained around those in Model 2, but with widened 95% CIs (Table 4).

After adjusting for age, sex, region, years of education, physical activity level, intended physical exercises, smoking status, alcohol use and daily energy intake (Model 2), magnesium from red meat, as well as from vegetables and fruit, was negatively associated with MetS risk, while magnesium from grains and potato was positively associated (*p* values for the trends < 0.01, < 0.01 and = 0.01, respectively). After further mutually adjusting for iron, zinc and magnesium from the same food sources (Model 3), the trends between magnesium from red meat and MetS risk, as well as between magnesium from vegetables and fruit and MetS risk, were no more statistically significant, while the trends between magnesium from grains and potato and MetS risk remained significant (*p* value for the trend < 0.01). The ORs for MetS risk across the quartiles of magnesium from red meat became closer to 1. The ORs for MetS risk across the quartiles of magnesium from grains and potato were larger than those in Model 2. The ORs across the quartiles of magnesium from vegetables and fruit remained around those in Model 2 but with widened 95% CIs (Table 4).

Sex differences were not observed in the associations of dietary sources of iron, zinc and magnesium with MetS in the current participants (Supplement Table S2).

## 4. Discussion

In the current study, we found that dietary iron, zinc and magnesium intakes were all associated with the risk of MetS independent of the high correlations between them. According to Chinese dietary reference intakes [22], iron, zinc and magnesium intakes were generally adequate on the basis of age-specific distribution among the current participants. One particularly notable finding was that iron intake among males exceeded more than 50% of the reference intake. We observed dietary iron, zinc and magnesium played different roles in the associations with MetS. Total dietary total iron was positively associated with the occurrence of MetS, while zinc demonstrated an inverse relationship in our study. These two findings were consistent with previous studies. The iron intake was high in the current participants. High consumption of dietary iron could be positively associated with abnormal metabolism [23]. A possible mechanism is that free iron has strong pro-oxidant properties and generates reactive oxygen species by participating in Fenton chemistry, which results in the induction of oxidative damage and apoptosis [24]. Zinc protects cells against oxidative stress damage, and a possible mechanism it that it inhibited proinflammatory cytokine expression, which in turn suppressed reactive oxygen species production. Our results were consistent with previous studies indicating that zinc intake shares a negative association with MetS [25]. It was beyond our expectation that dietary magnesium was positively associated with MetS in the current analysis. This finding was inconsistent with most previous studies which have suggested an inverse association between magnesium and MetS. It is possible that other factors in food highly correlated with magnesium were confounders in the relationship between dietary magnesium and MetS.

In terms of different types of iron, we discovered that haem iron was negatively correlated with the risk of MetS and that non-haem iron coincided with the trend of total iron with MetS in the current study population. Though Western studies indicate that haem iron or red meat is positively associated with MetS, the Chinese plant-based diet can probably be attributed to the difference between the results of Western studies and ours. Although non-haem iron has been widely acknowledged to have less bioavailability than haem iron, other nutrients in food, like ascorbic acid, can enhance non-haem iron absorption. Indeed, dietary non-haem iron was correlated with serum ferritin in our study population. This was consistent with the results of studies focusing on Chinese population [26].

Grain and potato, vegetables and fruit, as well as animal meat were the major food sources of iron, zinc and magnesium among the participants from China’s mega cities. With regard to the red meat source, iron, zinc and magnesium were no more statistically associated with MetS after mutually adjusting for the rest two minerals. However, we considered higher zinc intake to be associated with lower MetS risk. The 95% CIs of the ORs for the quartiles of zinc compared with the reference quartile were widened and no more statistically significant after further adjusting for iron and magnesium (Model 3 vs. Model 2), likely due to the high correlations between iron, zinc and magnesium, rather than statistical judgment without bias.

With regard to grain and potato sources, magnesium was positively associated with MetS, iron was not associated, and zinc was inversely associated among the study population. Much higher risks of MetS were in observed in association with the increasing quartiles of magnesium intake after further adjusting iron and zinc from grains and potato (Model 3 vs. Model 2). Magnesium was suggested to be a protective factor against MetS in previous studies [27]. From our understanding, the association of dietary magnesium from grains and potato with MetS was possibly confounded by other elements or even heavy metals in the same food sources highly correlated with magnesium. Studies in environmental science indicate that an unreasonable use of fertilizer would result in the continuous accumulation of heavy metals in soils, which would lead to excessive heavy metals entering into crops through their roots [28]. Magnesium is a common element in chemical fertilizers and fertilizers are essential to improve soil productivity for further increases in grain yields, especially in a country with large population, like China. It was a guess that food rich in magnesium is probably under the fertilizer usage, but food is in fact impacted by exposure to soils with high concentrations of heavy metals. The relationship between dietary magnesium and MetS in the current study was possibly distorted by other factors closely related with magnesium in grains and potato. This hypothesis warrants further study.

With regard to vegetable and fruit sources, all the three minerals were no more associated with MetS after mutual adjustment. Vegetable and fruit consumption has been widely acknowledged as a favorable factor for the prevention of MetS. We predicted that iron and magnesium intakes were actually correlated with MetS. The 95% CIs of the ORs were widened and no more statistically significant after mutually adjusting for iron, zinc and magnesium, but the ORs remained close to those in Model 2 (Model 3 vs. Model 2). The widened 95% CIs were probably influenced by the high correlations between iron, zinc and magnesium rather than statistical judgment without bias.

A limitation of this study is the methodology used to assess dietary intake. Since 3-day 24-h dietary recalls were used to obtain food consumption information, the accuracy of dietary intake was mostly dependent on the participants’ recall and estimations. Furthermore, the high correlations between iron, zinc and magnesium caused collinearity when simultaneously including these three variables in the same regression model. This probably caused bias in the statistical results. The associations between iron, zinc or magnesium with MetS might be also influenced by other nutrients possibly contributing to the development of MetS. Moreover, we cannot avoid the possibility that non-response bias, and other unknown confounding factors, might influence the results of the risk factor analysis. Finally, it was logical to hypothesize that dietary intake influenced the body’s metabolism, but causal inferences between dietary intake and MetS cannot be drawn due to the cross-sectional nature of the current study.

## 5. Conclusions

Dietary iron and magnesium was positively but zinc was inversely associated with the risk of MetS in China’s mega cities. The positive association of dietary magnesium with MetS could be a result confounded by other factors correlated with magnesium in grains and potato, which warrants further study.

## Figures and Tables

**Table 1:** Characteristics of the participants in the study population.

Characteristics	All	Male	Female
*n* (%)	5323 (100.00)	2386 (44.83)	2937 (55.17)
Age, %			
18–44 years	26.22	24.33	27.72
45–59 years	38.56	37.15	39.68
60+ years	35.22	38.52	32.60
Smoking Status, %			
Never smoker	68.18	36.69	93.36
Former smoker	5.72	10.48	1.91
Current smoker	26.10	52.83	4.73
Region, %			
Central City	41.02	40.31	41.59
Fringe Area	27.73	27.10	28.24
Outskirt	31.25	32.59	30.18
Physical Activity Level, %			
Sedentary	80.75	73.37	86.65
Moderate	15.41	21.05	10.90
Vigorous	3.84	5.58	2.45
Intended Physical Exercises, %	24.97	24.24	25.54
Alcohol Use, %			
lifetime abstainers	71.08	50.49	87.53
non-heavy drinkers	22.11	36.13	10.90
infrequent heavy drinkers	3.80	7.12	1.16
frequent heavy drinkers	3.01	6.26	0.41
Years of Education, %				
under 6 years	8.40	4.26	11.72
6 years	20.92	20.75	21.05
9 years	33.41	34.73	32.36
12 years	22.26	23.14	21.56
15 years	8.23	9.33	7.36
over 15 years	6.78	7.80	5.96
Dietary Intake, means ± SD			
Energy, kcal/day	1771.11 ± 582.02	1921.61 ± 610.97	1650.80 ± 527.90
Total Iron, mg/day	17.65 ± 6.18	18.82 ± 6.26	16.72 ± 5.96
Haem iron, mg/day	1.24 ± 0.95	1.32 ± 0.96	1.18 ± 0.93
Non-haem iron, mg/day	16.41 ± 5.81	17.50 ± 5.93	15.54 ± 5.57
Iron from Red Meat, mg/day	1.18 ± 1.19	1.34 ± 1.31	1.05 ± 1.06
Iron from Grain and Potato, mg/day	6.73 ± 3.36	7.44 ± 3.48	6.17 ± 3.14
Iron from Vegetables and Fruit, mg/day	3.88 ± 2.83	3.95 ± 2.89	3.82 ± 2.78
Total Zinc, mg/day	9.17 ± 3.21	9.89 ± 3.29	8.60 ± 3.03
Zinc from Red Meat, mg/day	1.52 ± 1.47	1.73 ± 1.62	1.35 ± 1.31
Zinc from Grain and Potato, mg/day	3.67 ± 1.68	4.06 ± 1.73	3.36 ± 1.58
Zinc from Vegetables and Fruit, mg/day	1.83 ± 1.62	1.86 ± 1.66	1.81 ± 1.59
Total Magnesium, mg/day	253.27 ± 98.74	267.15 ± 98.93	242.17 ± 97.19
Magnesium from Red Meat, mg/day	10.66 ± 9.83	12.19 ± 10.97	9.44 ± 8.63
Magnesium from Grain and Potato, mg/day	102.38 ± 58.88	111.26 ± 59.10	95.28 ± 57.74
Magnesium from Vegetables and Fruit, mg/day	77.65 ± 57.35	78.31 ± 58.86	77.12 ± 56.11
Metabolic Syndrome, %	34.49	29.48	38.49
Metabolic Syndrome’s components, %			
Elevated blood pressure	53.55	57.09	50.72
Elevated waist circumference	42.53	32.17	50.82
Elevated fasting glucose	34.81	36.00	33.86
Elevated triglycerides	29.06	30.98	27.52
Reduced HDL-C	36.15	25.27	44.86

**Table 2:** Partial correlation coefficients for dietary iron, zinc and magnesium intakes in 5323 participants from the China Nutrition and Health Survey (CNHS) and Shanghai Diet and Health Survey (SDHS) ^1,2^.

	Total Iron	Haem Iron	Non-haem Iron	Zinc	Magnesium
Total Iron	1	0.42	0.99	0.71	0.82
Haem Iron		1	0.31	0.54	0.31
Nonhaem Iron			1	0.68	0.82
Zinc				1	0.79
Magnesium					1

^1^ Correlations were adjusted for age and sex. ^2^ All coefficients are significant at the 0.05 level.

**Table 3:** Odds ratios (ORs) (95% CI) for metabolic syndrome (MetS) according to the quartiles of dietary iron, zinc and magnesium intakes (mg/day) in 5323 participants from CNHS and SDHS ^1, 2^.

		Quartiles of Dietary Iron, Zinc or Magnesium (mg/day), ORs (95% CI)				
		Q1	Q2	Q3	Q4	*p*-Value for Trend
	n	1330	1331	1331	1331	
**Total Iron**						
		< 13.14	(13.14, 16.73)	(16.73, 21.41)	≥ 21.41	
	Model 1	Reference	1.23(1.05, 1.45)	1.24(1.06, 1.46)	1.17(1.00, 1.37)	0.03
	Model 2	Reference	1.27(1.07, 1.51)	1.31(1.08, 1.59)	1.32(1.05, 1.64)	0.02
	Model 3	Reference	1.35(1.10, 1.65)	1.47(1.15, 1.88)	1.60(1.21, 2.11)	0.01
**Haem Iron**						
		< 0.61	(0.61, 1.05)	(1.05, 1.66)	≥ 1.66	
	Model 1	Reference	0.82(0.70, 0.96)	0.72(0.61, 0.84)	0.67(0.57, 0.78)	< 0.01
	Model 2	Reference	0.81(0.69, 0.96)	0.69(0.58, 0.82)	0.68(0.57, 0.82)	< 0.01
	Model 3	Reference	0.84(0.71, 1.00)	0.75(0.62, 0.91)	0.78(0.63, 0.96)	0.03
**Non-haem Iron**						
		< 12.21	(12.21, 15.50)	(15.50, 19.96)	≥ 19.96	
	Model 1	Reference	1.34(1.14, 1.57)	1.33(1.13, 1.56)	1.23(1.05, 1.45)	< 0.01
	Model 2	Reference	1.39(1.17, 1.65)	1.42(1.17, 1.72)	1.35(1.08, 1.69)	< 0.01
	Model 3	Reference	1.46(1.19, 1.79)	1.54(1.21, 1.96)	1.53(1.16, 2.02)	< 0.01
**Zinc**						
		< 6.87	(6.87, 8.69)	(8.69, 11.19)	≥ 11.19	
	Model 1	Reference	1.01(0.86, 1.17)	0.85(0.73, 1.00)	0.80(0.68, 0.94)	0.01
	Model 2	Reference	0.95(0.81, 1.13)	0.80(0.66, 0.96)	0.69(0.55, 0.86)	< 0.01
	Model 3	Reference	0.76(0.63, 0.92)	0.55(0.44, 0.69)	0.46(0.35, 0.61)	< 0.01
**Magnesium (mg/day)**						
		< 182.98	(182.98, 235.03)	(235.03, 304.34)	≥ 304.34	
	Model 1	Reference	0.97(0.83, 1.13)	0.86(0.73, 1.00)	0.80(0.68, 0.94)	< 0.01
	Model 2	Reference	1.08(0.91, 1.29)	1.31(1.09, 1.58)	1.13(0.91, 1.41)	0.03
	Model 3	Reference	1.11(0.90, 1.36)	1.42(1.12, 1.81)	1.32(0.99, 1.75)	0.02

^1^ Model 1 was a crude model which included no covariate. Model 2 was adjusted for age, sex, region, years of education, physical activity level, intended physical exercises, smoking status, alcohol use and daily energy intake, which included the variables mentioned ahead as covariates in the regression models. Model 3 was adjusted for the covariates in Model 2 and additionally mutually adjusted for iron, zinc and magnesium (e.g., when focusing on the relationship between dietary iron and MetS risk, dietary zinc and magnesium were included in the regression models), which included the variables mentioned above as covariates in the regression models. ^2^ Hierarchical logistic regression models were applied to identify the trends between dietary iron, zinc or magnesium intakes and MetS risk. The levels (1, 2, 3 and 4) of the dietary intake quartiles and other covariates mentioned above were as the independent variables and the occurrence of MetS as the dependent variable in the regression models.

**Table 4:** ORs (95% CI) for MetS according to the quartiles of dietary iron, zinc and magnesium intakes (mg/day) in 5323 participants from CNHS and SDHS, stratified by food sources ^1, 2^.

			Quartiles of Dietary Iron, Zinc or Magnesium (mg/day), ORs (95% CI)				
			Q1	Q2	Q3	Q4	*p*-Value for Trend
	*n*		1330	1331	1331	1331	
**Dietary Source of Red Meat**							
	**Iron**						
			< 0.35	(0.35, 0.91)	(0.91, 1.65)	≥ 1.65	
		Model 1	Reference	0.80(0.69, 0.93)	0.76(0.65, 0.89)	0.65(0.56, 0.76)	< 0.01
		Model 2	Reference	0.79(0.67, 0.93)	0.81(0.68, 0.96)	0.74(0.62, 0.88)	< 0.01
		Model 3	Reference	0.81(0.53, 1.25)	0.96(0.58, 1.60)	1.03(0.59, 1.82)	0.47
	**Zinc**						
			< 0.45	(0.45, 1.18)	(1.18, 2.21)	≥ 2.21	
		Model 1	Reference	0.85(0.73, 0.99)	0.73(0.62, 0.85)	0.65(0.56, 0.76)	< 0.01
		Model 2	Reference	0.84(0.72, 0.99)	0.77(0.65, 0.91)	0.74(0.62, 0.88)	< 0.01
		Model 3	Reference	0.97(0.59, 1.60)	0.70(0.39, 1.26)	0.71(0.38, 1.34)	0.21
	**Magnesium**						
			< 3.33	(3.33, 8.53)	(8.53, 15.60)	≥ 15.60	
		Model 1	Reference	0.79(0.68, 0.93)	0.83(0.71, 0.96)	0.62(0.53, 0.72)	< 0.01
		Model 2	Reference	0.81(0.68, 0.95)	0.85(0.72, 1.00)	0.71(0.60, 0.85)	< 0.01
		Model 3	Reference	1.01(0.64, 1.59)	1.20(0.71, 2.03)	0.97(0.54, 1.74)	0.29
**Dietary Source of Grain and Potato**							
	**Iron**						
			< 4.45	(4.45, 6.14)	(6.14, 8.26)	≥ 8.26	
		Model 1	Reference	1.25(1.06, 1.47)	1.52(1.29, 1.78)	1.63(1.39, 1.92)	< 0.01
		Model 2	Reference	1.32(1.11, 1.57)	1.72(1.44, 2.05)	2.08(1.71, 2.53)	< 0.01
		Model 3	Reference	1.04(0.82, 1.32)	1.09(0.81, 1.46)	1.19(0.84, 1.67)	0.77
	**Zinc**						
			< 2.55	(2.55, 3.38)	(3.38, 4.49)	≥ 4.49	
		Model 1	Reference	1.11(0.95, 1.31)	1.32(1.13, 1.55)	1.27(1.08, 1.48)	< 0.01
		Model 2	Reference	1.14(0.96, 1.35)	1.45(1.21, 1.72)	1.63(1.34, 2.00)	< 0.01
		Model 3	Reference	0.74(0.60, 0.92)	0.68(0.52, 0.88)	0.59(0.43, 0.81)	0.01
	**Magnesium**						
			< 62.55	(62.55, 89.87)	(89.87, 125.86)	≥ 125.86	
		Model 1	Reference	1.37(1.16, 1.61)	1.78(1.51, 2.10)	2.02(1.71, 2.37)	< 0.01
		Model 2	Reference	1.46(1.22, 1.74)	2.08(1.74, 2.48)	2.60(2.14, 3.16)	< 0.01
		Model 3	Reference	1.69(1.35, 2.13)	2.57(1.95, 3.39)	3.26(2.36, 4.50)	< 0.01
**Dietary Source of Vegetables and Fruit**							
	**Iron**						
			< 2.15	(2.15, 3.54)	(3.54, 5.51)	≥ 5.51	
		Model 1	Reference	0.86(0.74, 1.01)	0.89(0.76, 1.04)	0.81(0.69, 0.95)	0.07
		Model 2	Reference	0.83(0.67, 1.03)	0.76(0.61, 0.95)	0.69(0.55, 0.87)	0.01
		Model 3	Reference	0.93(0.67, 1.30)	0.88(0.59, 1.32)	0.77(0.48, 1.24)	0.72
	**Zinc**						
			< 0.82	(0.82, 1.38)	(1.38, 2.36)	≥ 2.36	
		Model 1	Reference	1.03(0.88, 1.20)	0.97(0.83, 1.14)	0.82(0.70, 0.96)	0.07
		Model 2	Reference	0.94(0.80, 1.11)	0.90(0.76, 1.07)	0.68(0.57, 0.81)	< 0.01
		Model 3	Reference	1.06(0.78, 1.43)	1.16(0.78, 1.72)	1.03(0.62, 1.69)	0.78
	**Magnesium**						
			< 38.60	(38.60, 63.36)	(63.36, 101.58)	≥ 101.58	
		Model 1	Reference	1.02(0.87, 1.19)	0.95(0.81, 1.11)	0.96(0.82, 1.12)	0.76
		Model 2	Reference	0.92(0.78, 1.08)	0.83(0.70, 0.98)	0.76(0.63, 0.90)	0.01
		Model 3	Reference	0.80(0.58, 1.09)	0.71(0.48, 1.05)	0.86(0.54, 1.36)	0.24

^1^ Model 1 was a crude model which included no covariate. Model 2 was adjusted for age, sex, region, years of education, physical activity level, intended physical exercises, smoking status, alcohol use and daily energy intake, which included the variables mentioned ahead as covariates in the regression models. Model 3 was adjusted for the covariates in Model 2 and additionally mutually adjusted for iron, zinc and magnesium (e.g., when focusing on the relationship between dietary iron and MetS risk, dietary zinc and magnesium were included in the regression models), which included the variables mentioned above as covariates in the regression models. ^2^ Hierarchical logistic regression models were applied to identify the trends between dietary iron, zinc or magnesium intakes and MetS risk. The levels (1, 2, 3 and 4) of the dietary intake quartiles and other covariates mentioned above were as the independent variables and the occurrence of MetS as the dependent variable in the regression models.

## Supplementary Materials

The following are available online at https://www.mdpi.com/2072-6643/12/3/659/s1, **Supplement Table S1.** Partial correlation coefficients between dietary iron intake and serum ferritin in the participants from CNHS and SDHS ^1^, **Supplement Table S2.** ORs (95% CI) for MetS according to the quartiles of dietary iron, zinc and magnesium intakes (mg/day) in male and female participants from CNHS and SDHS ^1^, **Supplement Table S3.** ORs (95% CI) for MetS according to the quartiles of dietary iron, zinc and magnesium intakes (mg/day) in male and female participants from CNHS and SDHS, stratified by food sources.
