# Supplementary material for: The Associations of Dietary Iron, Zinc and Magnesium with Metabolic Syndrome in China’s Mega Cities
Source: Nutrients. 2020 Feb 28;12(3):659. doi: 10.3390/nu12030659 (PMC7146276; doi:10.3390/nu12030659)
Supplement: Supplementary file 1 [file nutrients-12-00659-s001.pdf]

Supplement Table 1. Partial correlation coefficients between dietary iron intake and serum ferritin in the participants from CNHS and SDHS[1]

Dietary intake	serum ferritin
Total Iron	0.05*
Haem Iron	-0.01
Nonhaem Iron	0.06*

[1] Correlations were adjusted for age and sex.

* Coefficients are significant at the 0.05 level.

Supplement Table 2. ORs (95% CI) for MetS according to the quartiles of dietary iron, zinc and magnesium intakes (mg/day) in male and female participants from CNHS and SDHS[1].

	**Quartiles of Dietary Iron, Zinc or Magnesium (mg/day), ORs (95% CI)**				
	**Q1**	**Q2**	**Q3**	**Q4**	***p*-Value for Trend**
**Male**					
n	596	597	597	596	
**Total Iron**					
	＜14.12	(14.12, 17.75)	(17.75, 22.59)	⩾22.59	
Model1	Reference	1.11(0.86, 1.43)	1.39(1.09, 1.78)	1.32(1.03, 1.69)	0.03
Model2	Reference	1.05(0.80, 1.39)	1.26(0.94, 1.70)	1.21(0.86, 1.70)	0.41
Model3	Reference	1.20(0.88, 1.65)	1.56(1.07, 2.28)	1.57(1.02, 2.41)	0.12
**Haem Iron**					
	＜0.66	(0.66, 1.15)	(1.15, 1.74)	⩾1.74	
Model1	Reference	0.97(0.76, 1.24)	0.92(0.72, 1.17)	0.92(0.72, 1.17)	0.87
Model2	Reference	0.89(0.68, 1.15)	0.74(0.56, 0.97)	0.75(0.57, 1.00)	0.11
Model3	Reference	1.02(0.78, 1.35)	0.92(0.68, 1.24)	0.98(0.70, 1.36)	0.88
**Nonhaem Iron**					
	＜13.15	(13.15, 16.43)	(16.43, 20.92)	⩾20.92	
Model1	Reference	1.33(1.04, 1.72)	1.47(1.14, 1.89)	1.38(1.07, 1.77)	0.02
Model2	Reference	1.29(0.98, 1.70)	1.38(1.02, 1.85)	1.27(0.90, 1.80)	0.17
Model3	Reference	1.49(1.09, 2.04)	1.60(1.10, 2.32)	1.50(0.98, 2.30)	0.06
**Zinc**					
	＜7.50	(7.50, 9.39)	(9.39, 11.94)	⩾11.94	
Model1	Reference	0.84(0.66, 1.07)	0.86(0.67, 1.09)	0.84(0.66, 1.07)	0.42
Model2	Reference	0.78(0.60, 1.01)	0.65(0.49, 0.88)	0.62(0.45, 0.87)	0.02
Model3	Reference	0.66(0.50, 0.88)	0.47(0.33, 0.66)	0.42(0.28, 0.62)	<0.01
**Magnesium (mg/day)**					
	＜196.93	(196.93, 250.88)	(250.88, 323.64)	⩾323.64	
Model1	Reference	1.00(0.78, 1.28)	1.38(1.08, 1.76)	1.21(0.94, 1.55)	0.02
Model2	Reference	0.91(0.70, 1.20)	1.20(0.90, 1.60)	1.10(0.78, 1.54)	0.26
Model3	Reference	0.99(0.73, 1.35)	1.36(0.94, 1.96)	1.31(0.85, 2.02)	0.18

**Female**					
n	735	734	734	734	
**Total Iron**					
	<12.42	(12.42, 15.63)	(15.63, 19.96)	≥19.96	
Model1	Reference	1.26(1.03, 1.56)	1.26(1.03, 1.56)	1.30(1.05, 1.60)	0.05
Model2	Reference	1.28(1.01, 1.62)	1.18(0.92, 1.52)	1.34(0.99, 1.80)	0.16
Model3	Reference	1.21(0.91, 1.61)	1.17(0.84, 1.63)	1.43(0.98, 2.09)	0.23
**Haem Iron**					
	<0.58	(0.58, 0.98)	(0.98, 1.55)	≥1.55	
Model1	Reference	0.86(0.70, 1.05)	0.68(0.55, 0.83)	0.59(0.48, 0.73)	<0.01
Model2	Reference	0.82(0.66, 1.02)	0.68(0.54, 0.86)	0.61(0.48, 0.78)	<0.01
Model3	Reference	0.82(0.66, 1.04)	0.69(0.53, 0.89)	0.63(0.47, 0.84)	0.01
**Nonhaem Iron**					
	<11.57	(11.57, 14.51)	(14.51, 18.50)	≥18.50	
Model1	Reference	1.28(1.04, 1.58)	1.41(1.14, 1.74)	1.37(1.11, 1.69)	0.01
Model2	Reference	1.34(1.06, 1.69)	1.38(1.08, 1.78)	1.48(1.10, 1.99)	0.03
Model3	Reference	1.30(0.98, 1.72)	1.42(1.03, 1.96)	1.62(1.12, 2.33)	0.08
**Zinc**					
	<6.47	(6.47, 8.08)	(8.08, 10.29)	≥10.29	
Model1	Reference	1.03(0.84, 1.27)	0.96(0.78, 1.18)	0.89(0.72, 1.10)	0.57
Model2	Reference	1.00(0.80, 1.26)	0.87(0.68, 1.11)	0.75(0.56, 1.01)	0.16
Model3	Reference	0.82(0.64, 1.06)	0.63(0.46, 0.85)	0.52(0.36, 0.74)	<0.01
**Magnesium (mg/day)**					
	<173.85	(173.85, 224.70)	(224.70, 289.78)	≥289.78	
Model1	Reference	1.24(1.00, 1.53)	1.42(1.15, 1.75)	1.36(1.10, 1.67)	0.01
Model2	Reference	1.23(0.97, 1.55)	1.33(1.03, 1.70)	1.20(0.89, 1.62)	0.15
Model3	Reference	1.28(0.97, 1.71)	1.54(1.10, 2.15)	1.45(0.98, 2.16)	0.10

Supplement Table 3. ORs (95% CI) for MetS according to the quartiles of dietary iron , zinc and magnesium intakes (mg/day) in male and female participants from CNHS and SDHS, stratified by food sources.

	**Quartiles of Dietary Iron, Zinc or Magnesium (mg/day), ORs (95% CI)**				
	**Q1**	**Q2**	**Q3**	**Q4**	***p*-Value for Trend**
**Male**					
n	596	597	597	596	
**Dietary Source of Red Meat**					
**Iron**					
	<0.43	(0.43, 1.06)	(1.06, 1.89)	≥1.89	
Model1	Reference	0.99(0.78, 1.26)	0.80(0.63, 1.02)	0.81(0.63, 1.03)	0.13
Model2	Reference	0.91(0.71, 1.18)	0.79(0.61, 1.02)	0.75(0.57, 0.97)	0.12

Model3	Reference	1.10(0.66, 1.82)	1.12(0.59, 2.15)	1.29(0.60, 2.77)	0.91
**Zinc**					
	＜0.55	(0.55, 1.37)	(1.37, 2.50)	⩾2.50	
Model1	Reference	0.93(0.73, 1.18)	0.83(0.65, 1.06)	0.77(0.60, 0.99)	0.17
Model2	Reference	0.85(0.66, 1.10)	0.81(0.63, 1.05)	0.70(0.53, 0.92)	0.08
Model3	Reference	0.75(0.36, 1.58)	1.03(0.43, 2.48)	0.92(0.35, 2.43)	0.46
**Magnesium**					
	＜4.03	(4.03, 9.99)	(9.99, 17.67)	⩾17.67	
Model1	Reference	0.98(0.77, 1.24)	0.83(0.65, 1.06)	0.75(0.59, 0.97)	0.08
Model2	Reference	0.94(0.73, 1.22)	0.75(0.58, 0.98)	0.70(0.53, 0.92)	0.03
Model3	Reference	1.09(0.54, 2.20)	0.67(0.29, 1.53)	0.59(0.24, 1.47)	0.16
**Dietary Source of Cereal and Potato**					
**Iron**					
	＜5.06	(5.06, 6.73)	(6.73, 8.98)	⩾8.98	
Model1	Reference	1.09(0.85, 1.41)	1.50(1.17, 1.92)	1.50(1.17, 1.92)	<0.01
Model2	Reference	1.24(0.94, 1.62)	1.66(1.27, 2.18)	1.87(1.39, 2.52)	<0.01
Model3	Reference	1.19(0.82, 1.72)	1.34(0.87, 2.07)	1.34(0.80, 2.22)	0.61
**Zinc**					
	＜2.87	(2.87, 3.77)	(3.77, 4.84)	⩾4.84	
Model1	Reference	0.96(0.75, 1.24)	1.14(0.89, 1.46)	1.12(0.88, 1.43)	0.45
Model2	Reference	0.97(0.74, 1.26)	1.23(0.94, 1.61)	1.33(0.98, 1.79)	0.09
Model3	Reference	0.61(0.44, 0.86)	0.57(0.39, 0.84)	0.47(0.29, 0.74)	0.01
**Magnesium**					
	＜69.08	(69.08, 96.83)	(96.83, 135.73)	⩾135.73	
Model1	Reference	1.08(0.83, 1.40)	1.62(1.26, 2.08)	1.77(1.38, 2.27)	<0.01
Model2	Reference	1.20(0.91, 1.58)	1.81(1.38, 2.38)	2.29(1.69, 3.09)	<0.01
Model3	Reference	1.42(0.98, 2.04)	2.22(1.45, 3.39)	3.03(1.86, 4.95)	<0.01
**Dietary Source of Vegetable and Fruit**					
**Iron**					
	＜1.95	(1.95, 3.27)	(3.27, 5.14)	⩾5.14	
Model1	Reference	0.80(0.58, 1.11)	0.75(0.55, 1.04)	0.62(0.45, 0.86)	0.04
Model2	Reference	0.77(0.55, 1.07)	0.69(0.49, 0.97)	0.58(0.40, 0.83)	0.02
Model3	Reference	0.96(0.57, 1.64)	0.90(0.46, 1.74)	0.66(0.31, 1.42)	0.56
**Zinc**					
	＜0.82	(0.82, 1.39)	(1.39, 2.39)	⩾2.39	
Model1	Reference	0.92(0.72, 1.17)	0.94(0.74, 1.20)	0.86(0.67, 1.10)	0.68
Model2	Reference	0.87(0.68, 1.13)	0.86(0.67, 1.12)	0.74(0.56, 0.97)	0.18
Model3	Reference	1.57(0.93, 2.63)	2.56(1.34, 4.90)	2.35(1.05, 5.24)	0.04
**Magnesium**					
	＜38.60	(38.60, 63.11)	(63.11, 104.58)	⩾104.58	
Model1	Reference	0.90(0.71, 1.15)	0.83(0.65, 1.07)	0.90(0.70, 1.15)	0.54
Model2	Reference	0.83(0.64, 1.07)	0.76(0.58, 0.98)	0.76(0.58, 0.99)	0.13
Model3	Reference	0.47(0.28, 0.78)	0.30(0.16, 0.57)	0.35(0.16, 0.75)	<0.01

**Female**					
n	735	734	734	734	
**Dietary Source of Red Meat**					
**Iron**					
	＜0.32	(0.32, 0.80)	(0.80, 1.50)	⩾1.50	
Model1	Reference	0.74(0.60, 0.91)	0.72(0.58, 0.88)	0.65(0.53, 0.80)	<0.01
Model2	Reference	0.77(0.62, 0.96)	0.78(0.63, 0.97)	0.75(0.60, 0.95)	0.04
Model3	Reference	1.06(0.54, 2.07)	1.16(0.55, 2.43)	1.28(0.56, 2.91)	0.91
**Zinc**					
	＜0.40	(0.40, 1.06)	(1.06, 1.97)	⩾1.97	
Model1	Reference	0.75(0.61, 0.92)	0.69(0.56, 0.85)	0.63(0.51, 0.78)	<0.01
Model2	Reference	0.75(0.60, 0.93)	0.77(0.62, 0.97)	0.73(0.58, 0.91)	0.02
Model3	Reference	0.51(0.24, 1.11)	0.53(0.23, 1.24)	0.51(0.20, 1.28)	0.40
**Magnesium**					
	＜2.86	(2.86, 7.85)	(7.85, 13.67)	⩾13.67	
Model1	Reference	0.79(0.64, 0.97)	0.73(0.60, 0.90)	0.62(0.51, 0.77)	<0.01
Model2	Reference	0.82(0.66, 1.02)	0.80(0.64, 1.00)	0.74(0.58, 0.93)	0.06
Model3	Reference	1.42(0.77, 2.61)	1.29(0.64, 2.60)	1.12(0.52, 2.43)	0.55
**Dietary Source of Cereal and Potato**					
**Iron**					
	＜4.05	(4.05, 5.57)	(5.57, 7.49)	⩾7.49	
Model1	Reference	1.23(0.99, 1.52)	1.72(1.39, 2.12)	1.98(1.60, 2.45)	<0.01
Model2	Reference	1.22(0.97, 1.54)	1.76(1.39, 2.23)	2.19(1.70, 2.84)	<0.01
Model3	Reference	1.22(0.89, 1.68)	1.41(0.94, 2.10)	1.39(0.88, 2.21)	0.42
**Zinc**					
	＜2.34	(2.34, 3.08)	(3.08, 4.04)	⩾4.04	
Model1	Reference	1.13(0.91, 1.40)	1.38(1.12, 1.70)	1.60(1.30, 1.97)	<0.01
Model2	Reference	1.04(0.83, 1.31)	1.35(1.07, 1.71)	1.78(1.37, 2.31)	<0.01
Model3	Reference	0.73(0.55, 0.98)	0.67(0.48, 0.95)	0.66(0.44, 1.01)	0.13
**Magnesium**					
	＜58.17	(58.17, 83.17)	(83.17, 118.28)	⩾118.28	
Model1	Reference	1.13(0.91, 1.41)	1.73(1.40, 2.14)	2.26(1.83, 2.80)	<0.01
Model2	Reference	1.14(0.90, 1.45)	1.79(1.42, 2.27)	2.53(1.95, 3.28)	<0.01
Model3	Reference	1.19(0.88, 1.62)	1.81(1.24, 2.62)	2.59(1.67, 4.00)	<0.01
**Dietary Source of Vegetable and Fruit**					
**Iron**					
	＜1.97	(1.97, 3.17)	(3.17, 4.88)	⩾4.88	
Model1	Reference	0.92(0.70, 1.20)	0.87(0.67, 1.15)	0.95(0.73, 1.25)	0.80
Model2	Reference	0.87(0.65, 1.17)	0.80(0.59, 1.08)	0.86(0.62, 1.17)	0.54
Model3	Reference	0.89(0.58, 1.38)	0.81(0.47, 1.39)	0.81(0.43, 1.54)	0.90
**Zinc**					
	＜0.80	(0.80, 1.33)	(1.33, 2.26)	⩾2.26	
Model1	Reference	1.09(0.88, 1.33)	1.08(0.88, 1.33)	0.86(0.69, 1.06)	0.09
Model2	Reference	1.00(0.80, 1.25)	0.97(0.77, 1.22)	0.65(0.51, 0.82)	<0.01

Model3	Reference	0.88(0.59, 1.31)	0.74(0.43, 1.24)	0.68(0.35, 1.33)	0.68
**Magnesium**					
	＜38.60	(38.60, 63.55)	(63.55, 99.63)	⩾99.63	
Model1	Reference	1.13(0.92, 1.39)	0.98(0.80, 1.21)	1.04(0.85, 1.29)	0.56
Model2	Reference	0.99(0.79, 1.25)	0.89(0.70, 1.12)	0.81(0.64, 1.03)	0.23
Model3	Reference	1.09(0.72, 1.65)	1.28(0.76, 2.15)	1.63(0.89, 2.99)	0.38
